# Foreign bodies in the tracheobronchial tree: clinical and radiological analysis of cases over a 50-year period

**DOI:** 10.36416/1806-3756/e20250514

**Published:** 2026-05-22

**Authors:** Isadora R Furlan, Antonio J M Cataneo, Erica N Hasimoto, Daniele C Cataneo

**Affiliations:** 1. Disciplina de Cirurgia Torácica, Departamento de Cirurgia e Ortopedia, Faculdade de Medicina de Botucatu, Universidade Estadual Paulista - UNESP - Botucatu (SP) Brasil.

**Keywords:** Foreign bodies, Respiratory aspiration, Bronchoscopy, Child

## Abstract

**Objective:**

To analyze cases of foreign body (FB) aspiration in the last 50 years, describing clinical presentations, radiological characteristics, extraction methods, complications, and surgical interventions.

**Methods:**

A documentary study was conducted by reviewing the medical records of patients diagnosed with FB aspiration in the 1976-2025 period at the São Paulo State University School of Medicine Hospital, located in the city of Botucatu, Brazil.

**Results:**

A total of 240 patients were analyzed; 60% were male, with a median age of 3 years (IQR, 1-10 years). A history consistent with FB aspiration was present in 82%. Radiological findings included atelectasis, in 30.7%; radiopaque FBs, in 22.6%; normal chest X-rays, in 19.2%; and hyperinflation, in 18.3%. Rigid bronchoscopy was performed in 92% of the patients, flexible bronchoscopy was performed in 3.5%, and laryngoscopy was performed in 1.3%. In 3%, the FB was spontaneously expelled. The sensitivity of clinical and radiological findings was 83% and 83.2%, respectively. Ten patients required surgery, and 79 developed complications.

**Conclusions:**

Any suspected FB aspiration should be investigated, even when clinical and radiological findings are negative. Prolonged FB retention in the bronchial tree can lead to severe and irreversible complications. Most FBs are radiolucent, predominantly consisting of grains or seeds. Endoscopic removal remains the mainstay of treatment, with only a minority of cases requiring surgery. Early intervention is essential to prevent complications.

## INTRODUCTION

Foreign body (FB) aspiration remains a significant global health concern, primarily affecting children under four years of age. Acutely, it poses a risk of asphyxiation,^1^ while chronically it may cause irreversible pulmonary damage.^2^ Unfortunately, many of these children are initially misdiagnosed with asthma because their early symptoms are nonspecific and the aspiration event is often unwitnessed by parents.^3^ ^,^ ^4^ Therefore, a high degree of clinical suspicion by the physician is essential for timely diagnosis and treatment. Many children present with normal chest X-rays,^2^ ^,^ ^5^ and the absence of a clear choking history may lead physicians to overlook FB aspiration. Even when a history of “choking” is present, it may not be properly valued during the first medical consultation.^2^ ^,^ ^5^ The most common presenting symptoms include coughing, choking, dyspnea, cyanosis, and wheezing.^6^ The classic triad of cough, noisy breathing, and unilateral decreased breath sounds occurs in approximately 40% of cases,^7^ although most patients present with one or more of these findings rather than the full triad. 

The highest incidence of FB aspiration is observed in children between zero and three years of age,^2^ with peak mortality occurring between one and two years.^8^ ^-^ ^11^ Boys represent the majority of cases, accounting for 51% to 73%.^2^ ^,^ ^6^ ^,^ ^12^ Children are particularly susceptible because of the lack of molars for proper food grinding; immature swallowing and glottic reflexes; a tendency to explore objects orally; and the fact that aspiration often occurs while running, playing, or crying.^13^ Studies have shown that 80-96% of aspirated FBs are radiolucent, primarily consisting of organic materials such as grains and seeds, while metallic objects represent a small proportion.^2^ ^,^ ^14^ The presence or absence of symptoms depends on FB size, FB location, and duration of impaction. Typically, three distinct clinical phases follow FB aspiration. The first is characterized by coughing and choking, sometimes accompanied by cyanosis, occurring immediately after aspiration. The second phase may include noisy breathing, stridor, or respiratory distress, although some patients become asymptomatic as the FB lodges and reduces airway reflexes. The third, also known as complication phase, is marked by obstruction, infection, pneumonia, atelectasis, and fever,^13^ which can progress to bronchiectasis and lung destruction if the FB remains unremoved.^2^ Although endoscopic extraction is the preferred treatment, some complicated cases require surgical intervention, such as bronchotomy and tracheostomy, for FB removal. Surgery may also be necessary during the chronic phase, when prolonged retention has caused irreversible parenchymal damage, requiring resection.^2^ ^,^ ^14^ The objective of the present study was to analyze cases of FB aspiration treated over a 50-year period in our thoracic surgery service, focusing on clinical presentations, radiological characteristics, methods of extraction, complications, and surgical interventions. 

## METHODS

All cases of FB aspiration were reviewed through analysis of all medical records from the 1976-2025 period in the archives of the Thoracic Surgery Service of the São Paulo State University School of Medicine Hospital, a tertiary referral center located in the city of Botucatu, Brazil. Data were collected on the types of FBs and their sites of impaction, as well as on patient clinical history and initial presentation. Information was also collected on prior medical care for suspected FB aspiration; clinical findings; radiological abnormalities; acute and chronic complications of FB aspiration and its treatment; and the need for surgical intervention. Bronchoscopies were performed by a thoracic surgeon specializing in respiratory endoscopy. Rigid bronchoscopies were performed under general anesthesia, whereas flexible bronchoscopies were performed under local anesthesia with sedation. FB removal was typically achieved using forceps or a basket extractor. Secretions were suctioned, and the bronchial tree was irrigated with saline. Before removal of the bronchoscope, the entire airway was carefully examined for residual FB fragments, particularly in cases involving seeds. When intense manipulation occurred, corticosteroids were administered for 48 h. In cases of purulent secretions, clinical or radiological evidence of infection, or prolonged FB retention, antibiotic therapy was also provided. When endoscopic removal was unsuccessful, a thoracotomy followed by bronchotomy was performed. In such cases, the bronchus was gently clamped proximal to the FB to prevent migration during manipulation. The bronchus was then opened transversely as close to the FB as possible, and, after extraction, it was closed with a few simple sutures. In patients with long-standing FB retention leading to fibroatelectasis, lobectomy was performed. Following lobectomy, the remaining bronchial tree was examined to ensure complete FB removal. Descriptive statistics were used to summarize patient data. Age was reported as median and interquartile range because of non-normal distribution. Categorical variables were expressed as frequencies and proportions. The sensitivity of clinical findings and imaging studies in detecting FBs was calculated. 

The present study was approved by the local research ethics committee on September 20, 2020 (Protocol no. 4288233). 

## RESULTS

### Patients

**Figure 1:**
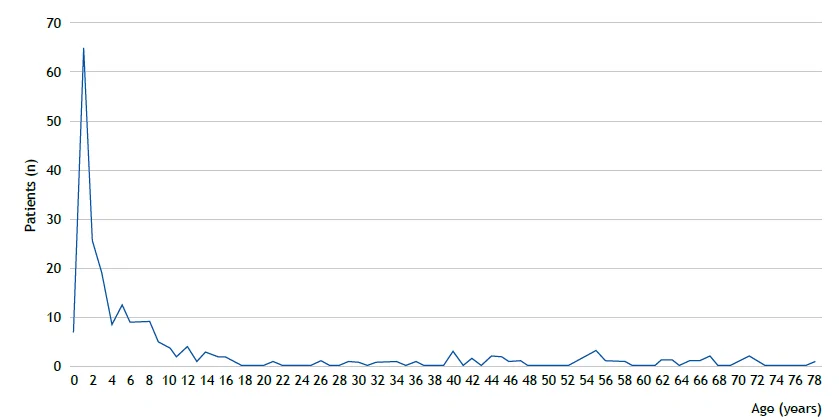
Distribution of patients by age.

Over the past 50 years, 240 patients presented to our thoracic surgery service with suspected FB aspiration. Of those, 60% were male. The median age was 3 years (IQR, 1-10 years), with 44% in the 0- to 2-year age bracket, 19% in the 3- to 6-year age bracket, 12.5% in the 7- to 10-year age bracket, 6% in the 11- to 16-year age bracket, and 18.5% being > 16 years of age (Figure 1). Eighty-two percent of the patients had a history consistent with FB aspiration. However, in 13% of those, this was ignored by the primary care physician because the radiograph was considered normal, despite their family members having reported FB aspiration. The remaining 18% had no history of aspiration and were admitted because of abnormal physical examination or imaging findings. 

Regarding symptoms, 68% presented with cough, 41% presented with dyspnea, 38.7% presented with cyanosis, 17.1% presented with wheezing, 12.7% presented with fever, and 3% were admitted to our hospital with acute respiratory failure. Regarding initial physical examination findings, 39.9% had reduced breath sounds, 21.5% had no changes, 17.1% had wheezing, 8.8% had rhonchi, 7% had crackles, and 3.9% had stridor. In 2 patients, because the FB was a whistle, it was audible during breathing. 

**Figure 2:**
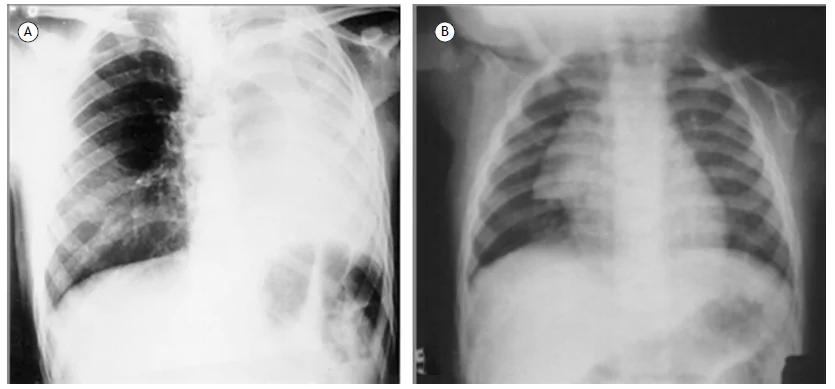
In A, a pen cap in the left main bronchus, leading to atelectasis of that lung. In B, a grain of corn in the emergence of the right upper lobe bronchus, leading to atelectasis of that lobe.

**Figure 3:**
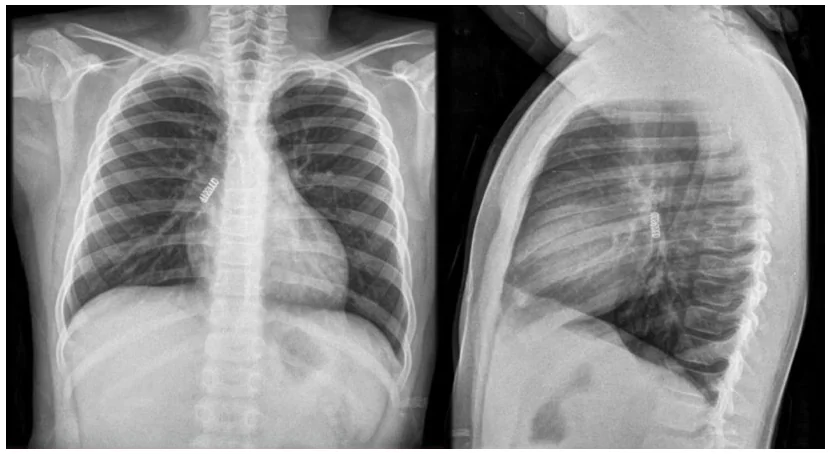
A pen spring (radiopaque foreign body) in the right bronchial tree.

**Figure 4:**
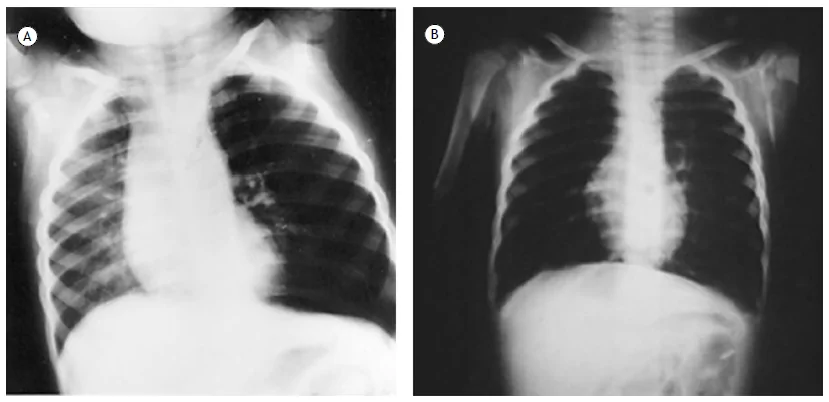
In A, a popcorn kernel in the left main bronchus, leading to hyperinflation of the left lung. In B, a bean kernel in the carina, leading to hyperinflation of both lungs.

Regarding plain chest X-ray findings, 6 patients (2.5%) did not undergo imaging, because they were admitted to our hospital either with a very typical history or in a serious state. Of the 234 patients who underwent examination, 30.7% presented with atelectasis (Figure 2), 22.6% presented with radiopaque FB (Figure 3), 19.2% presented with normal findings, 18.3% presented with hyperinflation or obstructive emphysema (Figure 4), 4.7% presented with consolidation, and 4.3% presented with volumetric reduction of one of the lung fields. 

Ninety-two percent underwent rigid bronchoscopy, 3.5% underwent flexible bronchoscopy, and 1.3% underwent direct laryngoscopy for FB removal. Three percent of the patients who underwent bronchoscopy expelled the FB before treatment was initiated, and endoscopic examination revealed signs of prior impaction, such as mucosal hyperemia, increased mucus production, and small lesions in the bronchial wall, but no FB was identified. Of the 240 patients, 10 required surgery: 6 required lobectomies because of destruction of lung lobes, and 4 required bronchotomies for removal of FBs that could not be removed by bronchoscopy. 

### Sensitivity of clinical history and sensitivity of radiological findings

Of the 240 patients who underwent procedures for FB identification and removal, 197 had a history of FB aspiration. Of those, 5 had no FB. Forty-three reported no FB aspiration, but 39 of those did have FBs. The sensitivity of clinical history was = 192/231 (i.e., 83%, with 39 cases [17%] being false negative). 

Of the 234 patients who underwent chest X-rays, 189 were abnormal, and FB was found in 188; only 1 had no FB found. Forty-five radiographs were considered normal, of which 38 had FBs. The sensitivity of chest X-rays was = 188/226 (i.e., 83.2%, with 38 cases [16.8%] being false negative). 

### Types and locations of FBs

**Figure 5:**
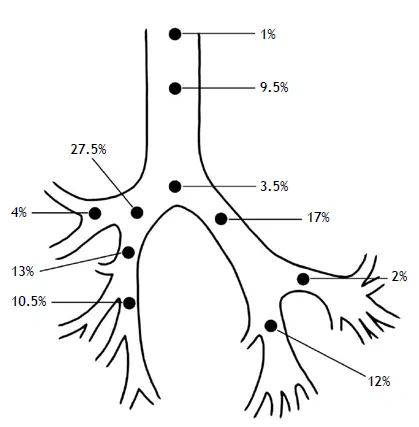
Proportions of foreign bodies in different locations of the tracheobronchial tree.

Grains, seeds, and nuts, such as peanuts, beans, coffee, corn, and fruit seeds, were the most common types of FBs (41.5%). Metallic materials, including screws, nails, pins, and other materials, accounted for 13%. Plastic parts, including various pen caps, accounted for 13%. Foods of animal origin, including beef, sausages, chicken bones, chicken cartilage, and fish bones accounted for 9.5%. Teeth and denture fragments accounted for 5.5%; 4.7% were toy parts; 2.1% contained fragments of plant fibers; 1.7% contained parts of tracheostomy tubes; and 9% were insects, stones, whistles, tracheostomy hygiene materials, gauze, surgical hemostatic material, and LED lamps. Fifty-five percent of the FBs were located on the right side, the majority being located in the main bronchus and intermediate bronchus (40.5%), and 31% were on the left, the majority being located in the main bronchus and lower lobe bronchus (29%); 9.5% were found in the trachea, 3.5% were found in the carina, and 1% was found in the larynx (Figure 5). 

### Length of time that the FB remained in the tracheobronchial tree and complications thereof

The time from onset of the clinical picture to appropriate treatment ranged from hours to more than one year, with most cases resolving within two days of onset. 

Ten patients (4.2%) required surgery: six lobectomies were performed because of destruction of lung lobes and four bronchotomies were performed to remove FBs that could not be removed by bronchoscopy. Seventy-nine patients (33%) developed the following complications: pneumonia, in 56; acute respiratory failure, in 7; destruction of lung lobes, in 6; pneumothorax and/or pneumomediastinum, in 4; bronchiectasis, in 3; bronchial stenosis after bronchotomy, in 1; and cardiorespiratory arrest, in 2, one of which died. The longer the FB remained in the tracheobronchial tree, the greater the number of complications. Those in whom the FB remained in the bronchial tree for up to two days had complications 8.5% of which were reversible, whereas those in whom the FB remained for three to 30 days had complications 34% of which were reversible and 4% of which were irreversible. Those in whom the FB remained from 30 days to years had complications 94% of which were reversible and 21% of which were irreversible (Table 1). 

**Table 1:** Period during which the foreign body remained in the tracheobronchial tree and complications thereof.

Period	N (%)	Complications
0-2 days	129 (54%)	8.5% reversible
3-30 days	77 (32%)	34% reversible 4% irreversible
30 days to > 1 year	34 (14%)	94% reversible 21% irreversible

## DISCUSSION

The findings of the present study show that children under four years of age are the most vulnerable to FB aspiration, being consistent with those of studies showing that children under five years of age account for more than 80% of cases.^2^ ^,^ ^12^ ^,^ ^14^ Males outnumber females, as observed in our series and in most reports.^2^ ^,^ ^12^ ^,^ ^14^ In the present study, grains and seeds were the most common FBs, with peanuts being the most common among those. This is unsurprising because peanuts are a common food in Brazil, consumed by children and adults. However, adults chew and swallow peanuts more easily, whereas young children, who have greater difficulty chewing, tend to keep fragments in their mouths longer, making aspiration more likely during crying or fright. Therefore, we strongly advise parents not to offer peanuts to young children. 

Zhijun et al.^15^ reported an exceptionally high number of peanut aspirations in a Chinese province, finding peanuts in 87% of 1,428 pediatric bronchoscopies. Other commonly aspirated seeds include beans and corn. Beans are typically aspirated raw, often when children play near their mothers during meal preparation. Regarding corn, unpopped popcorn kernels, which children may attempt to eat, and common feed corn can be aspirated. Plastic materials are usually small toy or pen parts that children disassemble and place in their mouths. Food fragments such as meat, fish spines, and bones, as well as teeth and dentures, are more common in adults, particularly those with chewing difficulties or those who eat hastily. Most FBs are organic materials.^2^ ^-^ ^4^ ^,^ ^6^ ^,^ ^8^ ^,^ ^11^ ^,^ ^14^ The type of aspirated organic FB varies with regional dietary habits. In Turkey, for example, watermelon seeds are the most common FB in summer.^16^

With respect to the impaction site, the present study showed predominance on the right side, consistent with the anatomy of the bronchial tree. Because the right main bronchus is wider and more vertically aligned with the trachea, aspirated material tends to lodge there. FBs impacted in the trachea or carina were generally large or denture fragments. Fortunately, these did not completely obstruct the airway. The larynx and trachea were the least affected in the present study, although they were associated with more severe conditions, such as complete obstruction and organ rupture. We observed severe cases in young children who aspirated raw beans that remained in the trachea; by the time they reached the hospital, the beans had swollen with humidity, eventually occluding the airway. Thus, seeds capable of turgor-driven expansion can cause serious complications and should be removed urgently. 

Although most patients present with a history consistent with FB aspiration, especially choking, a significant proportion of cases are initially overlooked.^2^ Following aspiration, coughing acts as a natural defense mechanism to expel the object. Depending on the bronchial diameter, FB type, and FB location, partial or complete airway obstruction may occur, causing stridor and cyanosis. Indeed, coughing, cyanosis, and noisy breathing are the most common symptoms and should raise suspicion of FB aspiration, even when no choking episode is witnessed. On physical examination, decreased breath sounds on the affected side are often detected. 

The sensitivity of clinical findings was evaluated by other authors,^17^ who found that a suggestive history of aspiration had 90% sensitivity. In our study, sensitivity was 83%, indicating that 17% of cases lacked a suggestive history (false negatives). Mothers often report that the child “swallowed” something rather than “aspirated” it, and when this report is accompanied by a normal radiograph, pediatricians may dismiss the diagnosis, an error observed in several of our cases. FB aspiration should always be suspected in recurrent or difficult-to-treat pneumonia. Studies show that in 42% of FB cases the diagnosis is not made at the first consultation.^18^

The most specific radiological findings are radiopaque FBs and segmental, lobar, or unilateral atelectasis, resulting from complete airway obstruction and alveolar air absorption. Hyperinflation can occur when partial obstruction allows air to enter during inhalation but prevents it from exiting during exhalation. This mechanism depends on intrathoracic pressure variations altering bronchial caliber. The frequency of hyperinflation in our study was lower than that reported by other authors,^14^ who observed obstructive emphysema in 60% of cases. However, their findings were based on inspiratory-expiratory radiographs or fluoroscopy, which are not routinely used, particularly in young children. Approximately 20% of imaging studies are normal^2^ ^,^ ^5^ ^,^ ^19^; thus, a normal radiograph with a suggestive history should not delay endoscopic evaluation.^2^ ^,^ ^17^ The sensitivity of radiography for FB detection in our study was 83.2%, with 17% false negatives. Therefore, a normal radiograph does not exclude FB aspiration. These findings reinforce that endoscopic evaluation should always be performed when clinical or radiological suspicion exists. 

CT has nearly 100% sensitivity for FB detection, as demonstrated in a systematic review including 18 studies and 4,178 patients.^20^ The authors suggested that CT could help avoid negative bronchoscopies. However, CT is not routinely used in our service because, aside from the very low number of negative bronchoscopies, a child with FB aspiration would require two anesthetics: one for CT and one for bronchoscopy. The same review concluded that although CT may be useful, it should not replace bronchoscopy. 

We observed that the longer the interval between FB aspiration and definitive treatment, the higher the number and severity of complications. Among those with FB retention for 30 days or more, almost all developed complications, and one fifth had irreversible damage. Perfusion scintigraphy studies also show that, 30 days after FB removal, perfusion abnormalities are more common in patients with prolonged FB retention.^21^

The most common complication in our study was pneumonia, while the most severe was pulmonary destruction, seen in six cases in which the FB remained for years within the bronchial tree. One patient died from asphyxiation caused by a tracheal FB, confirming that although tracheal FBs are less common, they can cause acute and fatal complications. Unfortunately, mortality from FB aspiration in children remains high.^22^ ^,^ ^23^

Regarding the technique used for FB removal, we prefer rigid bronchoscopy. Most authors recommend rigid bronchoscopy with optical guidance, as it allows direct airway access, excellent visualization, continuous oxygen and topical anesthetic administration, and passage of forceps for FB extraction. Some authors, however, have reported good results using flexible bronchoscopy even in children under two years of age.^24^ ^-^ ^26^

When endoscopic removal is not feasible, which is rare, surgical procedures such as bronchotomy and tracheotomy may be required. Surgery is also indicated in cases of lobar destruction. Early suspicion and prompt treatment are essential, as FB removal is a relatively simple procedure with low complication rates, while diagnostic delay can lead to serious and potentially irreversible outcomes. 

In conclusion, FB aspiration continues to be a common and potentially life-threatening condition, especially in children under four years of age. Clinical suspicion should remain high whenever a sudden episode of coughing, choking, or respiratory distress occurs, even when radiological findings are normal. Most aspirated FBs are organic and radiolucent, such as seeds and grains, and may remain undetected for long periods. Early diagnosis and prompt endoscopic removal are essential to avoid severe complications, including pneumonia, atelectasis, and bronchiectasis. Rigid bronchoscopy remains the procedure of choice for diagnosis and treatment. Surgery should be reserved for cases in which endoscopic removal fails or when chronic FB retention has led to irreversible pulmonary damage. 
